# Generation and Characterization of Mouse Models of C3 Glomerulonephritis With CFI D288G and P467S Mutations

**DOI:** 10.3389/fphys.2021.649801

**Published:** 2021-06-03

**Authors:** Hui Song, Mingchao Zhang, Xue Li, Feng Xu, Difei Zhang, Xiaodong Zhu, Jiong Zhang, Weisong Qin, Shaolin Shi, Jiqiu Wen

**Affiliations:** ^1^National Clinical Research Center for Kidney Diseases, Affiliated Jinling Hospital, Medical School of Nanjing University, Nanjing, China; ^2^Department of Nephrology, Guangdong Provincial Hospital of Chinese Medicine, Guangzhou, China

**Keywords:** complement factor I, amino acid variations, C3 glomerulopathy, mouse model, lipopolysaccharides

## Abstract

C3 glomerulopathy (C3GP) is a disease entity caused by abnormality of the complement alternative pathway (AP) and characterized by C3 deposition in glomeruli. Many variations or mutations of complement factors are believed to underlie the susceptibility to C3GP, but there is a lack of experimental evidence. We have recently reported a patient with C3 glomerulonephritis (C3GN) and compound heterozygosity of two novel variations in the complement factor (CFI). Here, we generated a mouse model to mimic the CFI variations for studying pathogenicity of CFI variations in C3GN development. We used the CRISPR/Cas9 system to make mutant mouse lines that carried D288G and P467S mutations in CFI, respectively, and crossed them to generate mice with compound heterozygosity of CFI D288G and P467S. The mice were all normal in either SPF (specific pathogen free) or regular environment. When treated with lipopolysaccharides (LPS), a bacterial endotoxin that mimics infection and sepsis, the mice developed albuminuria, kidney function impairment, and C3 glomerular deposition at levels comparable with the wild-type mice. The mice with other genotypes concerning CFI D288G and P467S were also tested in parallel. Unexpectedly, we found that the D288G homozygotes all developed severe mesangial deposition of C3 in the LPS model, indicating that CFI D288G variation was involved in the C3 deposition, a key feature of C3GN. The mouse lines generated in the present study can be used to further study the role of CFI variations in C3GN development; in addition, they may be used to screen and test infections and environmental factors capable of triggering C3GN.

## Introduction

C3 glomerulopathy (C3GP) which included Dense Deposit Disease (DDD) and C3 glomerulonephritis(C3GN) depending on the location of electronic density is a rare disease characterized by predominant C3 deposition in glomeruli and membranoproliferation in glomeruli (Fakhouri et al., 2010). Dysregulation of complement alterative pathway (AP) plays an important role in the pathogenesis of C3GP (Pickering et al., 2013; Schena et al., 2020), and the genetic variations or mutations in complement regulators have been implicated in AP dysregulation. For instance, homozygous or heterozygous mutations in the regulatory complement proteins factor H (CFH), factor I (CFI), C3, MCP (CD46), complement Factor B, and CFHRs are found in patients with C3GP (Smith et al., 2019). In addition to C3GP, AP abnormality is also implicated in thrombotic microangiopathy (TMA), including atypical hemolytic uremic syndrome (aHUS), as suggested by mutations in C3, CFI, CD46, and CFH found in patients with aHUS (Feitz et al., 2018). AP is constitutively active and serves as an immune surveillance and effector system operating in circulation and on cell surfaces. The activity of AP is tightly regulated to prevent the damage of self-cells in the body.

Although the variations/mutations in the complement regulators are believed to predispose the carriers to the C3GP and TMA, there are very few animal models that mimic the variations and prove their roles in the disease development, particularly for the single missense variations. Such animal models are important because the variations/mutations identified in the complement regulators in C3GP patients are not necessarily responsible for the diseases. First of all, the genetic defects in the complement-related genes have been identified only in a portion (∼25%) of the patients with C3GP (Bu et al., 2016; Smith et al., 2019); secondly, the functional consequences of the defects (especially those of single missense mutation) on the proteins have essentially not been tested; and thirdly, other known and unknown factors (e.g., variants of other genes, autoantibodies, etc.) may co-exist with the identified variations/mutations of complement regulators and actually interfere with the AP and cause the C3GP independently of the variations/mutations in the complement regulators (Ozaltin et al., 2013; Noris et al., 2019).

We have recently reported a case of C3GN combined with TMA in renal allograft (Wen et al., 2018). The gene test showed that this patient has two novel CFI gene variations, c.848A > G in exon 6 and c.1339C > T in exon 11, which resulted in D283G in the Ldlra domain (low-density lipoprotein receptor domain class A) and P447S in the trypsin-like serine protease domain, respectively. The c.848A > G allele was from his father and c.1339C > T from the mother. These two novel CFI variants presumably underlie the development of C3GN/MTA in the patient, which was likely triggered by a lung infection (Wen et al., 2018).

In the present study, we generated the CFI D288G and P467S compound mutations in mice that mimic the CFI variations in the patient. We found that these mice did not develop spontaneous C3GP and responded to LPS treatment similarly to wild-type mice concerning proteinuria, C3 glomerular deposition, circulating C3, and mesangial expansion. Mice with other genotypes of D288G and P467S mutations exhibited similar responses except for D288G homozygotes that had a severe C3 deposition, mesangial expansion, and reduced circulating C3, indicating that D288G affects CFI activity. These mouse models can be used for C3GN and MTA research and for screening and testing infections and environmental agents that can trigger C3GN.

## Materials and Methods

### Generation of CFI D288G and P467S Mutations in Mice Using CRISPR/Cas9

To create a CFI D288G mutant model using CRISPR/Cas9-mediated gene editing, we acquired from Ensembl^1^ the Cfi genome information, Cfi-001 ENSMUST00000077918, which has 14 exons, with the ATG start codon in exon 1 and TGA stop codon in exon 14 (Supplementary Figure 1). The Cfi gene targeting for CFI D288G and P467S was designed based on this information. To make CFI D288G point mutation in mice, two sgRNAs targeting the intron 6-7 and Exon 6 of Cfi gene were, respectively, constructed and transcribed *in vitro*. The donor vector with the Cfi-D288G fragment was designed and prepared. sgRNA sequences: 5′ sgRNA (5′–3′): ACCAATACAAGTGTAATGGTG; PAM: AGG; and 3′ sgRNA (5′–3′): ACATATGTGTGATGTGCACG; PAM: TGG. For P467S mutation, sgRNAs and donor vector were similarly prepared. For each mutation, the corresponding Cas9 mRNA, sgRNA, and donor were co-injected into mouse zygotes, and the zygotes were transferred into the oviduct of pseudopregnant ICR females at 0.5 dpc. The F0 mice were born 19∼21 days after implantation, and those with desired mutations were identified by sequencing the PCR product from mouse tail DNA. The F0 mice which had a copy of the point mutation of D288G or P467S in CFI were identified by genotyping following the method described below (Supplementary Figure 2). Next, the F0 mice were crossed with C57BL/6J mouse to generate heterozygotes. These two mouse lines were crossed (CFI-D288G^+/m^ x CFI-P467S^+/m^ to generate 1) CFI-D288G^m/m^ homozygotes, 2) CFI-D288G^+/m^ heterozygotes, 3) CFI-P467S^m/m^ homozygotes, 4) CFI-P467S^+/m^ heterozygotes, 5) CFI-D288G^+/m^;P467S^+/m^ compound heterozygotes (cHet) mice for further studies. The wildtype pups obtained from the crosses were used as controls. The generation of the mouse models and their breeding were conducted in the mouse facility of the Nanjing Biomedical Research Institute of Nanjing University. The treatment, sample collection, and sacrifice of the mice were performed in the mouse analysis laboratory at Jinling Hospital, Nanjing Univeristy School of Medicine.

### Thermal Cycling Condition of PCR

Most PCR amplifications were performed with a regular condition: incubated at 95°C/5 min, followed by 35 cycles of 95°C/30 s, 55–60°C/30 s, 72°C/5 min for 35 cycles, and then held at 10°C. For primer 1, 3, 5, and 7 (Supplementary Table 1), touch-down PCR was performed with following thermal cycling condition: 95°C/5 min, followed by 20 cycles of 98°C/30 s, 65°C/30 s (−0.5°C each cycle), and 72°C/45 s, and followed by another 20 cycles of 98°C/30 s, 55°C/30 s, and 72°C/45 s for 35 cycles, and then 72°C/5 min.

### LPS Treatment of Mice

LPS treatment was performed following our previous study (Lang et al., 2019). Briefly, LPS (Sigma Aldrich, MO, United States) was dissolved in saline at a concentration of 1 mg/ml, and was injected intraperitoneally at the dose of 10 mg/kg. Twenty-four hours later, the second injection at the same dose was performed. Twelve hours after the second injection, the spot urine samples and kidney biopsies were collected from the mice for further analyses.

### Measurement of Urinary Albumin/Creatinine Ratio

We measured the urinary albumin and creatinine levels using the ELISA kit, Albuwell M (Exocell, Philadelphia, United States), and the QuantiChrom^TM^ Creatinine Assay Kit (Bioassay systems, CA, United States), respectively, following the manufacturer’s instructions. Urinary albuminuria levels were expressed as albumin/creatinine ratio (uACR, μg/mg).

### Animal Sacrifice, Perfusion, and Kidney Tissue Collection

Mice were briefly anesthetized by inhaling Isoflurane in a chamber, followed by intraperitoneal injection of ketamine/xylazine hydrochloride solution (dose of ketamine, 100 mg/kg; xylazine, 10 mg/kg body weight). When mice were completely anesthetized, they were killed by perfusion with PBS buffer through left ventricle after blood collection from orbital sinus. Kidneys were then excised and cut into two halves, each of which was embedded in OCT compound and snap frozen with liquid nitrogen for immunofluorescence staining, and in 4% paraformaldehyde (PFA) followed by paraffin embedding for PAS staining, respectively.

### PAS Staining and Pathological Scoring

We used the same method for PAS staining as described previously (Wu et al., 2015). Glomerular mesangial expansion was scored as follow: 0, no any expansion; (1) 1–10% glomeruli with mild expansion; (2) 11–25% glomeruli with moderate expansion; (3) 26–50% glomeruli with severe expansion; (4) 50% glomeruli being sclerotic. Twenty glomeruli were examined and scored for each mouse. A pathologist who was blinded to the sample identities performed the evaluation and scoring.

### Immunofluorescence Staining

Sections of 5-μm thickness of mouse frozen kidney tissues were blocked with 10% FBS and incubated with primary antibodies labeled with FITC, rabbit polyclonal anti-C3, IgG, and IgA (DAKO, United States). The images were captured under the Leica microscope (DM5000B). For quantification of C3 deposition, we used ImageJ (the National Institutes of Health, United States) to determine the intensity of staining in glomeruli. For IgG and IgA, a pathologist blinded to the sample identities examined the glomeruli and scored staining intensity by 0 (no staining), 1 (weak staining), and 2 (positive staining).

### Measurement of Serum C3 of the Mice

Serum C3 levels were measured using the ELISA kit for serum C3 measurement (the Cloud-clone Inc, Wuhan, China, SEA861Mu). We performed the measurement following the manual instruction.

### Measurement of Serum Creatinine, BUN, and Albumin of the Mice

We performed serum creatinine, BUN and albumin measurements for the mice following the methods that we have described previously (Wu et al., 2015).

### CD3 and CD68 Immunohistochemical Staining of Kidney Tissues

Immunohistochemical staining of CD3 (Novocastra, NCL-L-CD3-565) and CD68 (Dako, Anti-Human CD68 clone PG-M1 M0876) was performed on mouse kidney sections following the method described (Wu et al., 2015).

### Statistical Analyses

The data are presented as the mean ± SD. The results were analyzed using GraphPad Prism 6 software (GraphPad Software Inc., CA, United States). The differences between two groups were analyzed using a two-tailed Student’s *t*-test. Based on whether the assumption of normal distribution and homogeneity of variance was met or not, ANOVA or Kruskal-Wallis test was used for statistical analyses, followed by *post hoc* Dunn’s test.

### Study Approval

The animals and experimental procedures were approved by the Institutional Animal Care and Use Committee of Jinling Hospital, Nanjing University School of Medicine, China (2015NZGKJ-057).

## Results

### Determination of Point Mutations to Be Made in Mouse CFI That Mimic the Patient’s Variations

**FIGURE 1:**
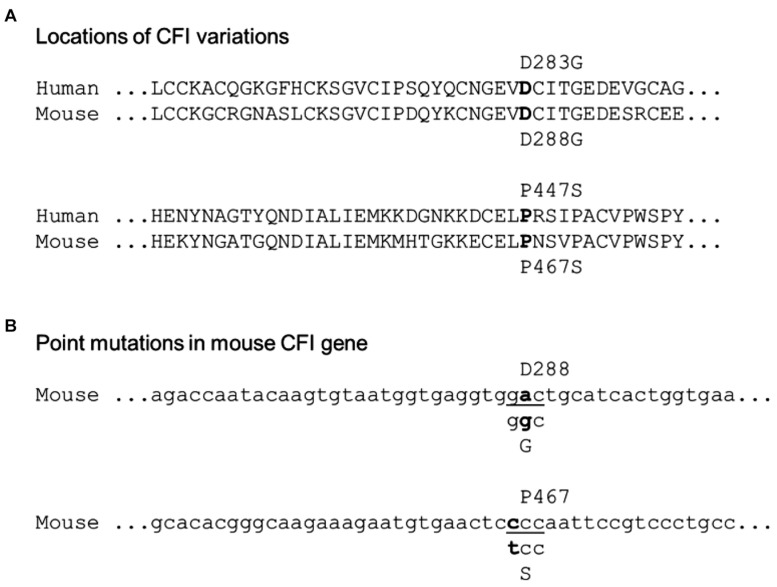
Localization of the patient’s CFI variations in mouse counterpart. **(A)** Alignment of human and mouse CFI amino acid sequences near the variations. The human D283 vs. mouse D288 and human P477 vs. mouse P467 are indicated. **(B)** Design of point mutations in CFI gene to be made in mice: CFI D288 codon “gac” to be swapped to “ggc” for glycine (G), and P467 codon “ccc” to “tcc” for serine (S).

To map the patient’s CFI variations to the mouse CFI genes, we aligned the amino acid residue sequences of human and mouse CFI (Figure 1A). The two amino acid residues of human CFI, D283, and P447, which were mutated in the patient, are conserved in mouse (D288, P467). The corresponding nucleotide sequence of mouse Cfi gene and the point mutations (c.848A > G and c.1339C > T) to be made according to the patient’s CFI are shown in Figure 1B. We followed the procedure described in the Methods to generate mice of various genotypes concerning CFI-D288G and -P467S mutations.

### CFI-D288G/P467S cHet Mice Were Normal

**FIGURE 2:**
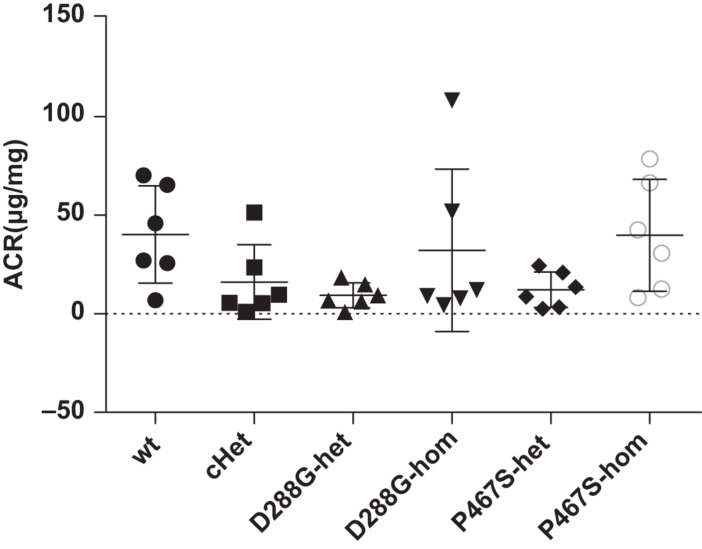
The CFI D288G/P467S cHet mice and the mice with other genotypes did not develop proteinuria spontaneously at the age of 12 weeks. Urinary albumin/creatinine ratio (μg/mg) was measured for the mice in the groups of mice as indicated, and they all had a normal level of urinary albumin. Wt, wildtype; cHet, compound D288G/P467S heterozygotes; D288G-Het, D288G heterozygotes; D288G-Hom, D288G homozygotes; P467S-Het, P467S heterozygotes; P467S-Hom, P467S homozygotes. *n* = 6 for all groups. No statistically significant differences were observed among the groups according to the Kruskal-Wallis test (*p* = 0.079).

The CFI-D288G/P467S cHet mice were born with the expected Mendelian ratio in the breeding, indicating that the compound heterozygosity of CFI-D288G/P467S did not cause embryonic lethality. These mice grew normally with a body weight comparable with wild-type littermates and had no observable abnormalities, e.g., albuminuria, as determined by albumin/creatinine ratio (ACR) of spot urine, at a specific pathogen free (SPF) environment. We also left the mice in a regular, non-SPF environment, allowing certain potential pathogens in the environment to trigger C3GN in the mice. However, after exposure to the regular environment for more than a month, the mice were still normal and did not develop albuminuria (the mice were 12 weeks old at the time). The mice with other genotypes, including D288G heterozygotes, D288G homozygotes, P467S heterozygotes, and P467S homozygotes, were also normal as shown by lack of proteinuria (Figure 2).

### CFI-D288G/P467S cHet Mice Responded to LPS Treatment Similarly to Wild-Type Mice

Patients with susceptibility to C3 and TMA can be fine under a normal condition, but can develop the disease with certain stimuli from environment, e.g., infections. Unfortunately, the exact factors capable of triggering the disease are not known. We then chose and tested LPS, an agent that mimics infection and sepsis, and hoped that it would induce C3GN in the CFI-D288G/P467S cHet mice.

**FIGURE 3:**
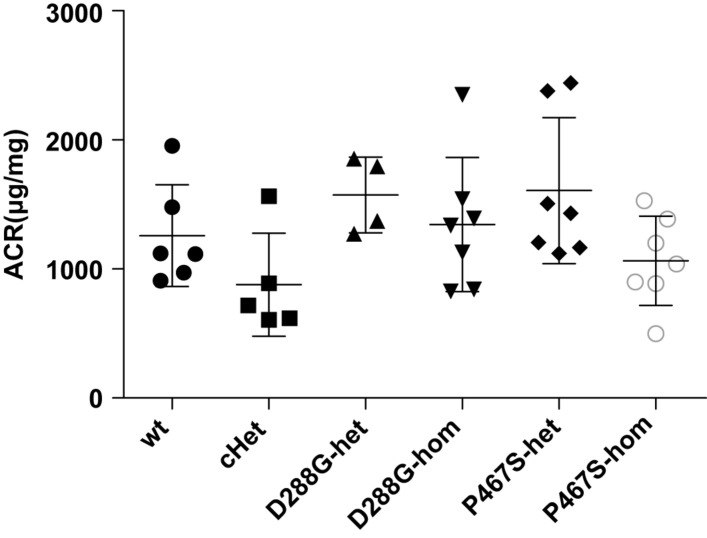
The albuminuria of mice of CFI-D288G/P467S cHet and other genotypes after LPS treatment. Thirty-six hours after the first 10 mg/kg LPS injection, spot urine samples were collected and subjected to ACR measurement. Wt, *n* = 6; cHet, *n* = 5; D288G-Het, *n* = 4; D288G-Hom, *n* = 7; P467S-Het, *n* = 7; and P467S-Hom, *n* = 7. ANOVA analysis was performed and showed no statistically significant differences among the groups (*p* = 0.071).

We found that all the mice developed proteinuria at the comparable levels (Figure 3). The success of the LPS model was further demonstrated by the increased serum BUN, serum creatinine and decreased serum albumin after LPS treatment (Supplementary Figure 3). cHet mice were expected to have a higher level of proteinuria because they mimicked the patient of C3GN. However, their proteinuria was comparable with that of wild-type and other mice. Consistently, the serum creatinine, BUN, and albumin levels of the cHet mice were also similar to wildtype and other mice (Supplementary Figure 4).

**FIGURE 4:**
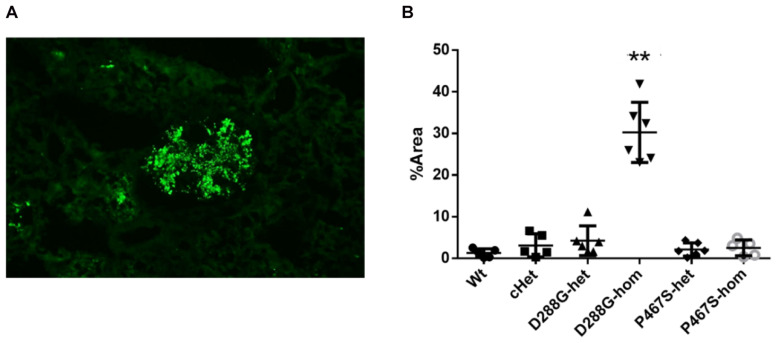
Examination of C3 deposition in the kidneys of the mice treated by LPS. **(A)** An example of C3 staining pattern in mesangium. **(B)** Quantification and comparison of C3 staining intensity in the mice in the 6 groups. Wt, *n* = 5; cHet, *n* = 5; D288G-het, *n* = 6; D288G-hom, *n* = 6; P467S-het, *n* = 6; P467S-hom, *n* = 5. **p = 0.0012 vs. wt according to the Kruskal-Wallis test with Dunn’s multiple comparisons.

**FIGURE 5:**
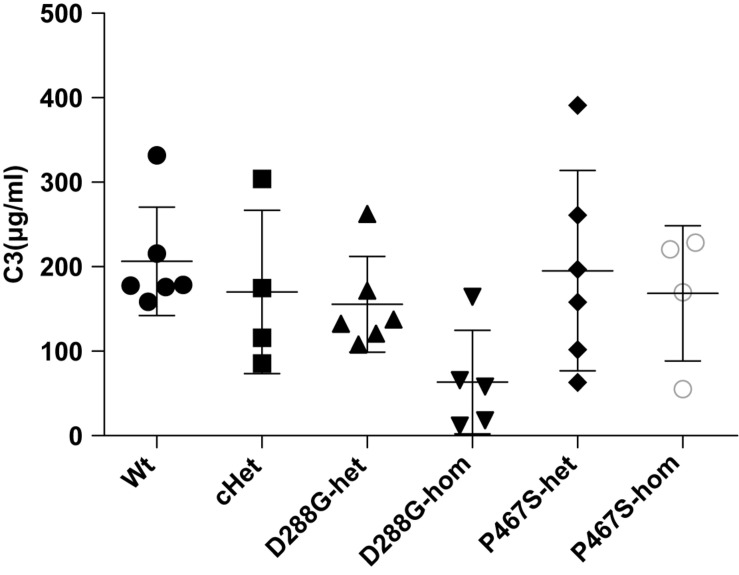
Comparisons of the circulating C3 levels of the groups of mice with different genotypes under the treatment of LPS. The serum C3 level of the D288G homozygote group was lower than that of the other groups, but the differences did not reach any statistical significance (*p* = 0.114) according to the ANOVA test. Wt, *n* = 6; cHet, *n* = 4; D288G-het, *n* = 6; D288G-hom, *n* = 5; P467S-het, *n* = 6; P467S-hom, *n* = 4.

**FIGURE 6:**
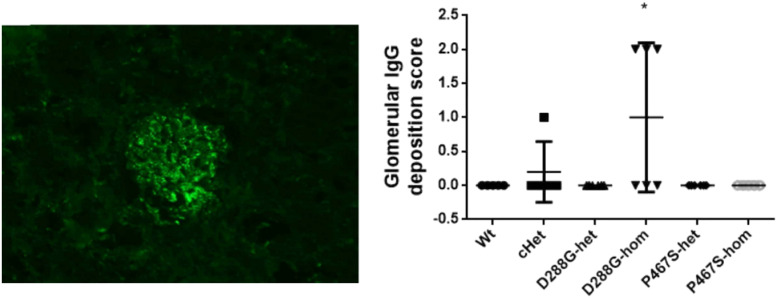
Examination of IgG deposition in the kidneys of the mice treated by LPS. An example of IgG staining pattern in a glomerulus (left), and the quantification and comparison of IgG intensity in the mice in the 6 groups. Wt, *n* = 5; cHet, *n* = 5; D288G-het, *n* = 6; D288G-hom, *n* = 6; P467S-het, *n* = 6; P467S-hom, *n* = 5. *p = 0.052 vs. wt according to the Kruskal-Wallis test.

We performed C3 immunofluorescence staining on the kidney sections of the mice, and found that cHet mice had a weak mesangial deposition of C3 at levels similar to wild-type mice (Figure 4). We also measured the circulating C3 in the mice and found that cHet mice had a similar level of serum C3 to that of wildtype mice (Figure 5). Additionally, we performed IgG and IgA staining, and found similar levels of IgG intensity between cHet and wild-type mice (Figure 6). IgA staining was negative for all of them (data not shown). In the inspection of inflammatory cells in the kidney sections stained with CD3 and CD68 antibodies, we did not find overt infiltration of T cells and macrophage cells in all the groups of mice (Supplementary Figure 5). No red blood cells were found in the sediment of urine of all the groups (data not shown), precluding hematuria in the mice. In the pathological examination of the kidneys, no thrombotic microangiopathy was found in any mice (data not shown).

### CFI-D288G Homozygotes Developed C3GN Phenotypes After LPS Treatment

In the above examinations, we noticed that CFI-D288G homozygotes had a C3 deposition in glomeruli that was much more severe than that of mice in the other groups in the LPS model. As shown in Figure 4, we performed semi-quantitative analysis of the staining intensity of each mouse using ImageJ and compared the results across the groups. We found that D288G homozygotes had a much higher intensity of C3 in glomeruli than the other mice (*p* = 0.0012 vs. wt, Figure 4). In the IgG staining, half of the D288G homozygotes were positive while the other half were negative. IgG staining was negative in all the mice in the other groups except that one mouse in the cHET group showed weak positive staining (Figure 5). C3 and IgG staining in tubules and blood vessels was minimal. In the serum C3 measurement, there was no difference between the groups (*p* = 0.114, Figure 6).

**FIGURE 7:**
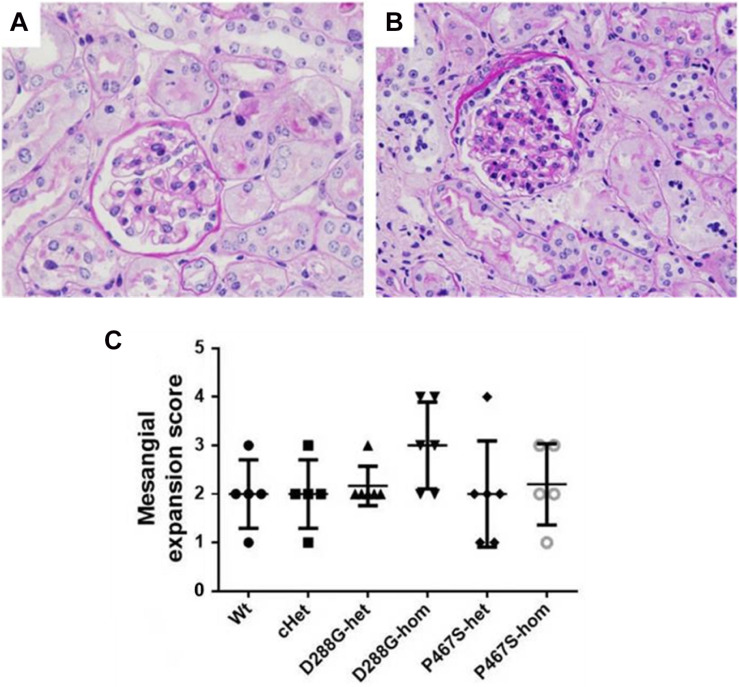
Mesangial expansion in the mice of CFI-D288G/P467S cHet and other genotypes after LPS treatment. **(A,B)** PAS staining showing normal glomeruli in mouse kidney **(A)** and a glomerulus with mesangial expansion **(B)** (x600). **(C)** Quantification of mesangial expansion in the 6 groups of mice. Wt, *n* = 5; cHet, *n* = 5; D288G-het, *n* = 6; D288G-hom, *n* = 6; P467S-het, *n* = 6; P467S-hom, *n* = 5. Kruskal-Wallis test was used for statistical analysis, showing no significance among groups (*p* = 0.307), although the D288G homozygote group tended to have a higher score than the other groups.

In the PAS staining of kidney sections, mild mesangial expansion was noted in some mice in each group (Figures 7A,B), however, there was no statistical difference in severity of mesangial expansion across the groups of mice (Figure 7C).

## Discussion

In the present study, we generated the first animal models of human CFI variants, and found that D288G and P467S compound heterozygous mice that mimicked the CFI variations in a previously reported patient did not develop spontaneous C3GN. With LPS treatment, the D288G/P467S cHet mice developed proteinuria, mesangial expansion and C3 deposition at levels similar to the mice of wild-type. Unexpectedly, the D288G homozygotes all had much more severe mesangial C3 deposition compared with the mice in the other groups. It is possible that with proper treatment, the D288G/P467S cHet mouse model of the patient’s CFI variations would also be induced to develop C3GN or MTA. Therefore, these mouse models can be used for further study of the role of the CFI variations in C3GN and MTA development. In addition, we propose that these mouse lines could be used as a tool for screening of infections and environmental agents that are capable of triggering C3GN and TMA in individuals with CFI variations, and the feasibility of such screening has been clearly demonstrated with the model of LPS which successfully induced C3GN phenotypes in the D288G homozygotes mice. The environmental agents can be various drugs, chemicals, pollutants and even food.

At present, a number of variations in CFI have been reported in patients with C3GP and aHUS (Fremeaux-Bacchi et al., 2004; Caprioli et al., 2006). Pathogenic variations of CFI are localized at several conserved domains, including the “low-density lipoprotein receptor domain class A” (Ldlra) and “the trypsin-like serine protease domain” (Tryp) (Servais et al., 2007; Leroy et al., 2011) (HGMD database)^2^. The two novel CFI variations (D283G and P447S) in the patient that we reported previously (Wen et al., 2018) are also localized to these two domains, respectively, and thus likely underlie the pathogenesis of C3GP and TMA in the patient. We therefore expected that the mice with the compound heterozygosity of D288G and P467S (cHet) as in the patient would develop C3GN and/or TMA. However, the cHet mice did not differ from the mice of wild-type and other genotypes essentially under normal conditions and LPS treatment.

Although we did not observe glomerular C3 deposition in the D288G/P467S cHet mice under the particular experimental conditions in this study, the potential of compound and heterozygous D288G and P467S variations to cause glomerular C3 deposition cannot be precluded. There might be several reasons for the failure of the cHet mice to develop glomerular C3 deposition in the LPS treatment. First of all, modeling complement variations in animals may not be easy if additional factors, which are absent in mice, are required for the onset of disease in patients. For instance, in humans, even the nonsense mutations that lead to CFI deficiency do not fully penetrate, leaving a portion of individuals with the mutations free of the disease (Fremeaux-Bacchi et al., 2004). In fact, most genetic variants have a low penetrance and require additional variations/mutations or other factors, e.g., thrombomodulin (Schena et al., 2020), to cause a disease. Secondly, mice may tolerate CFI deficiency better than humans due to certain differences of the complement system between mice and humans (Pouw et al., 2015). For example, CFI deficiency can cause C3GN in humans (Servais et al., 2007), whereas CFI knockout does not cause spontaneous glomerulonephritis and proteinuria, but does result in mesangial C3 deposition and expansion in a portion of the knockout mice (Rose et al., 2008). Thirdly, an appropriate trigger of the disease might be critical for the onset of the disease for a given variation. LPS may not best mimic the lung infection that caused C3GP in the patient that we reported previously (Wen et al., 2018), thus requiring the right pathogens to be identified and used to treat the cHet mice for glomerular C3 deposition. Unfortunately, we did not have the information of how the infection occurred to the patient.

Interestingly, in contrast with the cHet and other genotypes, D288G homozygotes developed clear and severe C3 deposition in mesangium in the treatment of LPS. This resembles the CFI knockout mice without LPS treatment (Rose et al., 2008), suggesting that D288G homozygosity and LPS acted in concert to alter complement alternative pathway, resulting in elevation of complement activity. Mutations in the Ldlra domain of Cfi have been found in patients with C3GP (Servais et al., 2007; Leroy et al., 2011), suggesting that the homozygous D288G mutation in the mice could impair CFI activity and be responsible for the increased severity of glomerular lesions in the D288G homozygote mice in LPS treatment. On the other hand, the D288G homozygotes did not exhibit a more severe proteinuria than other mice after LPS treatment. It is known that LPS can directly act on podocytes as a ligand for TLR4, resulting in podocyte cytoskeletal injury and massive proteinuria in mice, and this direct proteinuria-inducing effect of LPS should be stronger than that of C3GP triggered by LPS, causing the D288G homozygote group to have similar proteinuria as that in the other groups.

Glomerular C3 deposition occurred in the D288G homozygotes but not the D288G/P467S cHet mice. We speculate that the D288G mutation may cause a greater loss of CFI activity than P467S mutation although further experiments are required to prove it. It is possible that D288G homozygosity, but not D288G/P467S cHet, reaches a threshold of CFI activity loss, which leads to C3GN development. Thus, the homozygous D288G mice could be a more sensitive model than D288G/P467S cHet and other genotypes in detecting C3GN-inducing factors. However, the cHet mice may have the potential to detect C3GN inducers as well. Further studies are required toward this direction.

Our present study has suggested that the CFI variations were responsible for the C3GN on the renal allograft of our patient. According to our study, we consider that he could receive a second renal transplantation. His transplanted kidney could be safe as long as he avoids infections and exposure to C3GN-inducing factors. If an infection or exposure does occur, an immediate and most effective treatment should be followed to minimize the activation of the C3 system to avoid damage to his renal allograft.

One limitation of the present study was the relatively low number of mice of each genotype that were employed for the studies. A larger number of mice for each group with different genotypes would reveal clearer and more convincing results in the study. Another limitation is that the time period for observing the spontaneous development of C3GN in the mice was not long enough. Spontaneous C3GN might be observed in old mice.

## Data Availability Statement

The original contributions presented in the study are included in the article/Supplementary Material, further inquiries can be directed to the corresponding author/s.

## Ethics Statement

The animal study was reviewed and approved by the Institutional Animal Care and Use Committee of Jinling Hospital, Nanjing University School of Medicine, China (2015NZGKJ-057).

## Author Contributions

JW conceived the study. JW, HS, MZ, JZ, and SS designed the experiments. HS, MZ, JW, DZ, XZ, FX, and WQ performed the experiments. SS, JW, HS, MZ, and XL interpreted data and wrote the manuscript. All authors have reviewed and approved the final version of the manuscript.

## Conflict of Interest

The authors declare that the research was conducted in the absence of any commercial or financial relationships that could be construed as a potential conflict of interest.

## Footnotes

1 www.ensembl.org

2 http://www.hgmd.cf.ac.uk/
