# Supplementary material for: Generation and Characterization of Mouse Models of C3 Glomerulonephritis With CFI D288G and P467S Mutations
Source: Front Physiol. 2021 Jun 3;12:649801. doi: 10.3389/fphys.2021.649801 (PMC8209374; doi:10.3389/fphys.2021.649801)
Supplement: Supplementary file 1 [file Data_Sheet_1.docx]

**Supplementary Materials**

Generation of mouse models of C3 glomerulopathy with CFI D288G and P467S mutations

Hui Song, Mingchao Zhang, Xue Li, Feng Xu, Difei Zhang, Xiaodong Zhu, Jiong Zhang, Weisong Qin, Shaolin Shi, Jiqiu Wen

**Table S1.** Primer sequences and product information

No. Primers Sequences GC% Tm Size Notes


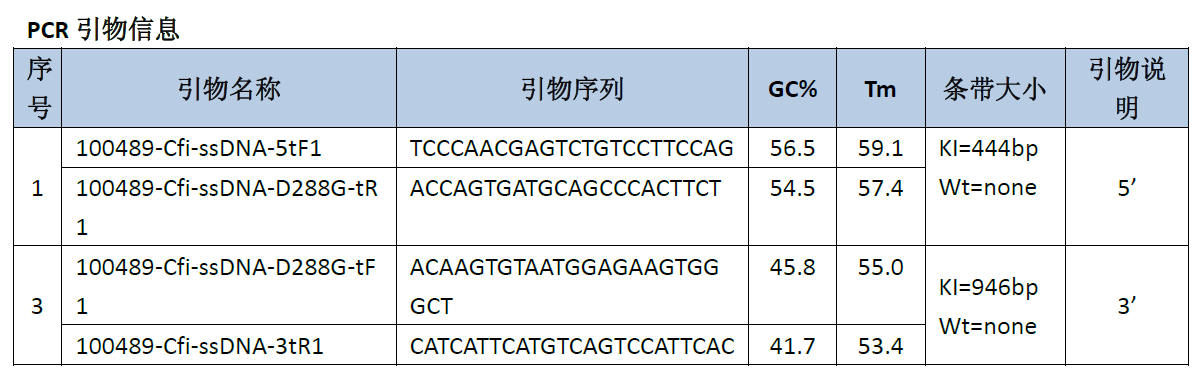


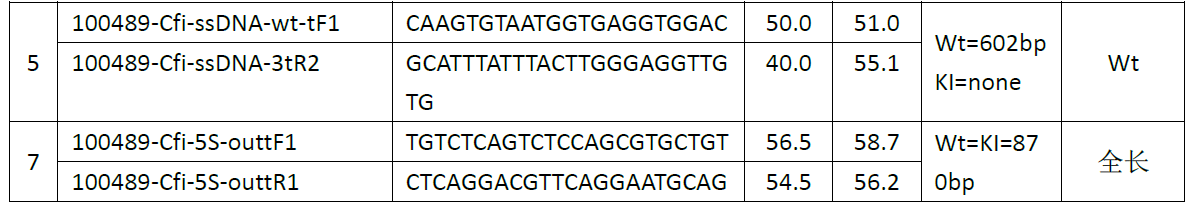


FL

Cfi-D288：


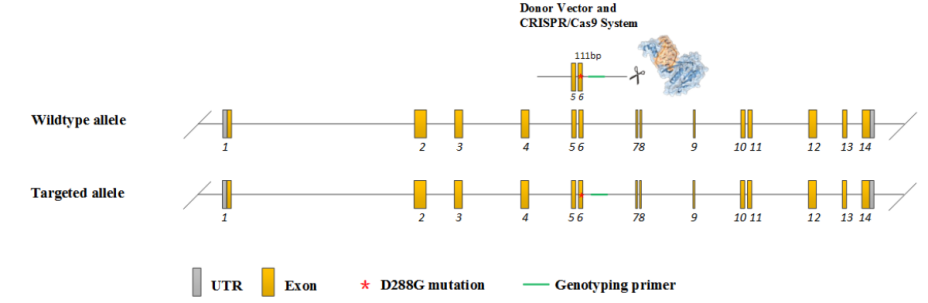


Cfi-P467S

**
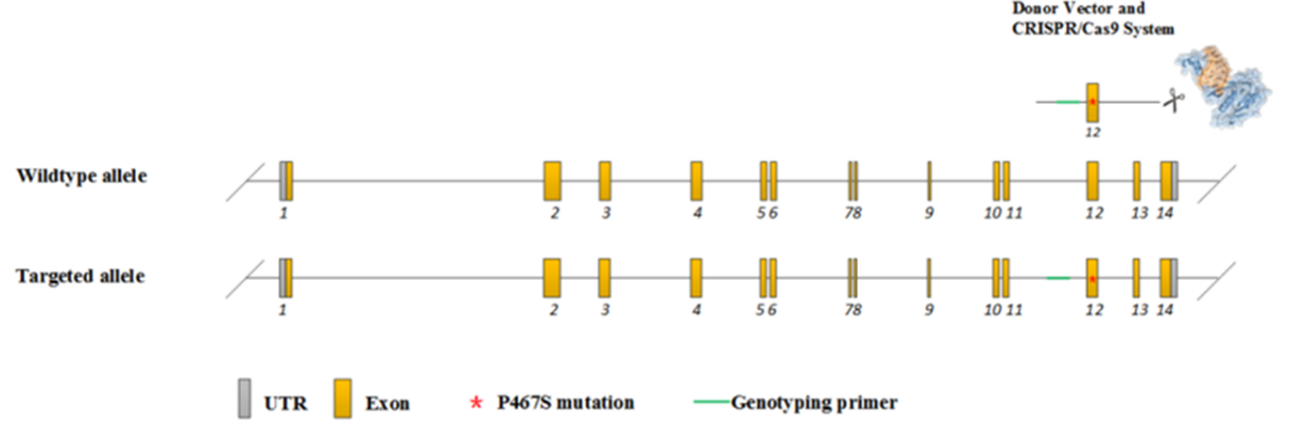
**

**Figure S1.**Genomic organization of mouse CFIgene. The location of each exon of CFI gene is shown, including the exons to be targeted for D288G (upper) and P467S (down)mutations as indicated by *.


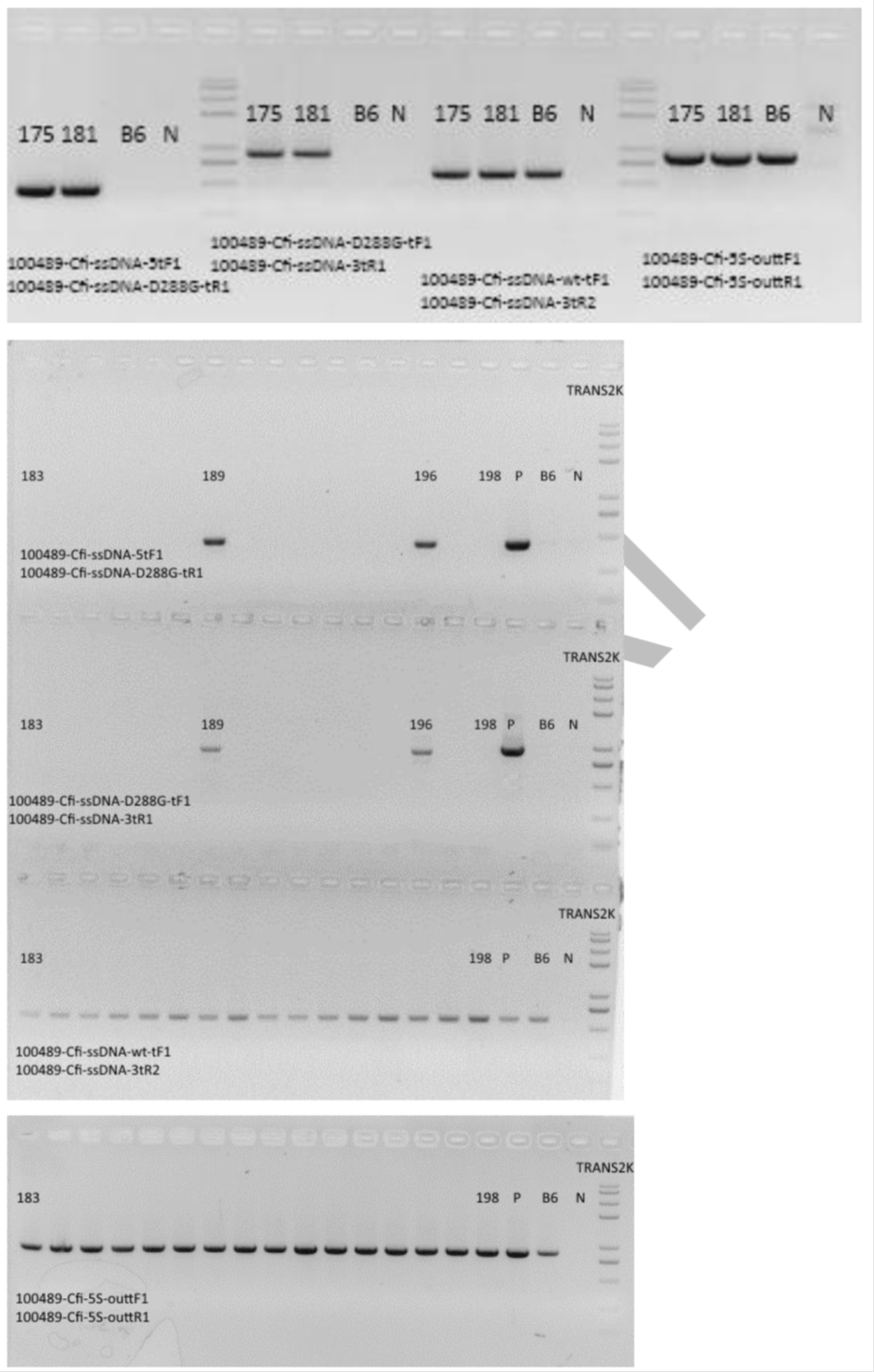


**Figure S2.**PCR genotyping results of the mice. The location of the primers are indicated in the Figure S1 as a green bar. The sequences of the primers are given in the Table S1 below.


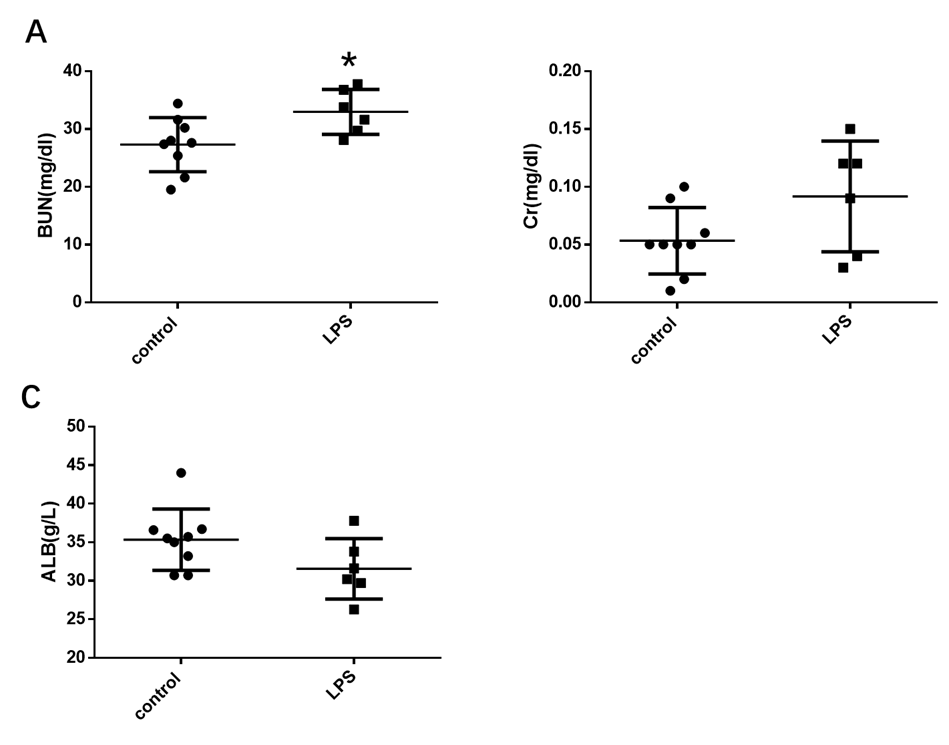


**Figure S3.**The kidney function of the wild-type mice after treatment with LPS. The serum levels of BUN (A) and creatinine (B) were increased (*p=0.029, and p=0.073, respectively), while serum albumin (C) decreased (p=0.093) after LPS treatment. Unpaired t-test was used for the statistical analyses.


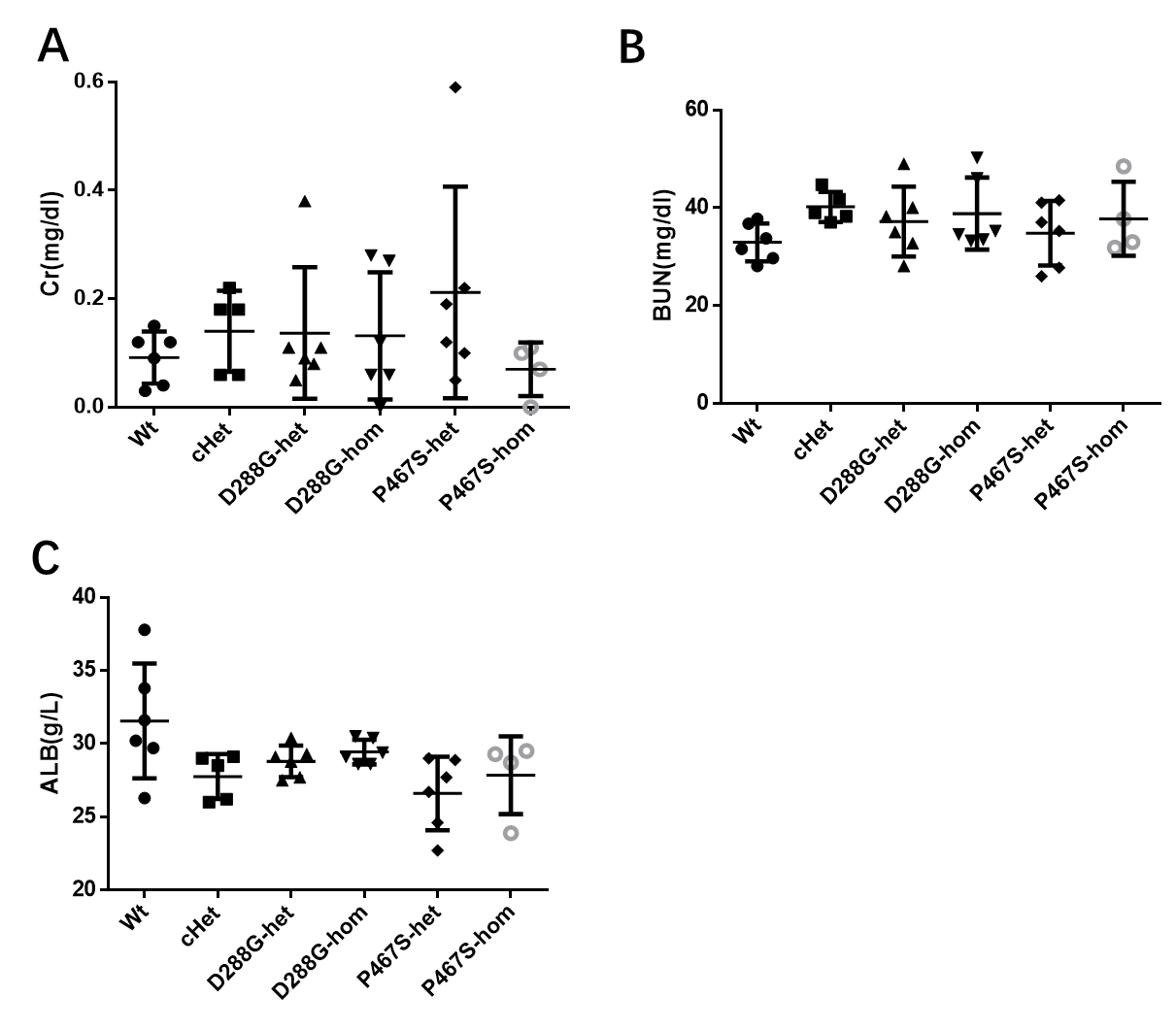


**Figure S4**. Comparisons of serum creatinine (A), BUN (B) and albumin (C). There was no any difference with statistical significance among the groups of mice according to theKluskal-Wallis test.


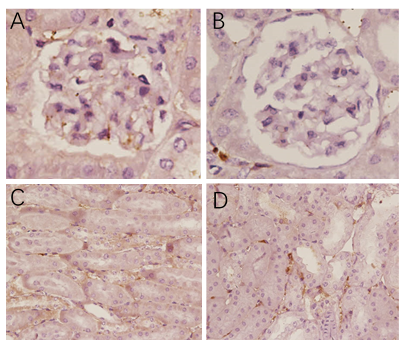


**Figure S5.** Inflammatory cells staining in kidney of the mice after LPS treatment. A, C. anti-CD3 staining of glomerular (A) and tubular areas (C), respectively; B, D. anti-CD68 staining of glomerular (B) and tubular areas (D). The results showed no significant infiltration of T cells and macrophage cells in the kidney.
